# Impact of Oxidative Stress on Age-Associated Decline in Oocyte Developmental Competence

**DOI:** 10.3389/fendo.2019.00811

**Published:** 2019-11-22

**Authors:** Hiroyuki Sasaki, Toshio Hamatani, Shintaro Kamijo, Maki Iwai, Masato Kobanawa, Seiji Ogawa, Kenji Miyado, Mamoru Tanaka

**Affiliations:** ^1^Department of Obstetrics and Gynecology, Keio University School of Medicine, Tokyo, Japan; ^2^National Center for Child Health and Development (NCCHD), Tokyo, Japan

**Keywords:** oxidative stree, mitochondrial dysfunction, antioxidants, oocyte aging, ER stress

## Abstract

Reproductive capacity in women starts to decline beyond their mid-30s and pregnancies in older women result in higher rates of miscarriage with aneuploidy. Age-related decline in fertility is strongly attributed to ovarian aging, diminished ovarian reserves, and decreased developmental competence of oocytes. In this review, we discuss the underlying mechanisms of age-related decline in oocyte quality, focusing on oxidative stress (OS) in oocytes. The primary cause is the accumulation of spontaneous damage to the mitochondria arising from increased reactive oxygen species (ROS) in oocytes, generated by the mitochondria themselves during daily biological metabolism. Mitochondrial dysfunction reduces ATP synthesis and influences the meiotic spindle assembly responsible for chromosomal segregation. Moreover, reproductively aged oocytes produce a decline in the fidelity of the protective mechanisms against ROS, namely the ROS-scavenging metabolism, repair of ROS-damaged DNA, and the proteasome and autophagy system for ROS-damaged proteins. Accordingly, increased ROS and increased vulnerability of oocytes to ROS lead to spindle instability, chromosomal abnormalities, telomere shortening, and reduced developmental competence of aged oocytes.

## Introduction

In reproductive health, a decrease in pregnancy rate and an increase in miscarriage rate are observed in women aged 35 and older. While the rate of live baby born per oocyte is 26% for women under age 35, it deteriorates at an accelerating pace and finally drops to 1% for those over age 42 (1). There has been extensive discussion about reduced quality of oocytes during aging, which is considered to cause chromosomal aberration in embryos and reduced embryonic developmental competence. The aging phenomena can be attributed to accumulated oxidative damage in somatic cells (2, 3), as mammalian oocytes also show increased ROS levels with age (4–6). The relationship between oxidative stress and oocyte deterioration has been well-investigated (7–10). Moreover, antioxidants such as melatonin and coenzyme Q10 have anti-aging effects on mouse oocytes by regulating mitochondrial functions and ROS levels in oocytes during reproductive aging (11–13). In this review, using the latest literature, we discuss the pathophysiology in which oocytes are more exposed and vulnerable to OS as they age, and the mechanisms by which oocyte quality is degraded.

### Age-Related Mitochondrial Dysfunction and Decreased Energy Production in Oocytes

Mitochondria are important organelles that act as sites of energy production in aerobic respiration. Mitochondria produce ATP during energy metabolism utilizing the redox reaction in the respiratory chain complex located on the inner membrane. At the same time, most of the superoxides produced *in vivo* are generated in mitochondria (14). ROS are constantly generated in the mitochondria of aerobic organisms, but are also eliminated by antioxidant enzymes in the mitochondria thus maintaining redox balance and homeostasis. However, when ROS are excessively generated or the antioxidant ability is reduced because of aging or disease, the redox balance is lost and ROS are accumulated. The increase in OS is closely related to mitochondrial dysfunction (15–17).

Mitochondrial dysfunction is indeed correlated with aging in somatic cells. For example, mammalian aging has been correlated with the accumulation of mtDNA deletions/mutations and reduced mitochondrial respiratory chain function (18, 19). mtDNA is vulnerable and easily mutated owing to its proximity to the respiratory chain producing ROS, its lack of protective histones, and deficiency of efficient repair mechanisms (20). Mitochondrial gene mutations in turn lead to reduced mitochondrial function, i.e., disturbance of the redox balance and OS. A mouse model called “mtDNA mutator mouse,” which harbors a D257A mutation in the exonuclease “proofreading” domain of DNA polymerase-γ (Polg) gene, exhibits a progeroid phenotype. Accelerated accumulation of mtDNA mutations and mitochondrial dysfunction lead to a systemic premature aging phenotype including reduced fertility (17, 21). Likewise, in oocytes, mitochondria are closely related to the decline of oocyte quality with age (22). Point mutations and deletions in the mtDNA in oocytes are also found with maternal aging (23–25). Moreover, embryos obtained from elderly women have an abnormally high copy number of mtDNA and such embryos do not implant (26–28). It can be presumed that a vicarious increase in mtDNA copy number via mitochondrial biogenesis may effectively compensate for heteroplasmic mtDNA mutations and mitochondrial dysfunction (15). However, considering the qualitative aspect, the relationship between mtDNA integrity in oocytes and ovarian aging is controversial. The accumulation of the 4977-bp deletion is related to age due to OS in somatic cells (29). Although the 4977-bp deletion also tends to increase with age in oocytes, the frequency of the mitochondrial mutation and IVF failure are not significantly correlated (30).

Morphological and ultrastructural changes of oocytes with age are also observed. They include increase of irregular mitochondria with changed matrix density, ooplasmic fraction of vacuoles and dilated smooth endoplasmic reticulum (ER) and Golgi complex (31, 32). *In vitro* matured eggs from aged mice did not have a cortical distribution of active mitochondria shown in those from young mice (33). Mitochondrial fusion and fission, adapting the size and shape, also play a critical role in maintaining the function in response to changes in the metabolic milieu. Mitofusin 1 and 2 mediate mitochondrial fusion. Oocyte-specific targeted deletion of either gene results in severely reduced oocyte quality with elevated ROS levels (34, 35). Moreover, mitochondrial fission maintains the competency of oocytes via multi-organelle rearrangement. A study on Dynamin-related protein 1 (*Drp1*) known as an oocyte-specific mitochondrial fission factor showed that mitochondria are highly aggregated with other organelles (e.g., ER and secretory vesicles) in oocytes from *Drp1*-deficient mice, resulting in impaired Ca^2+^ signaling and meiotic resumption. Oocytes from aged mice also showed a decrease in *Drp1*-dependent mitochondrial fission and defective organelle morphogenesis, which is similarly observed in *Drp1* KO oocytes (36). Since *Drp1* is also recruited by OS and plays a protective role (37), the decline in oocyte quality with age may be associated with a decrease in *Drp1* responsiveness to OS (38).

Mitochondrial dysfunction, which is caused by or causes OS, induces chromosomal non-disjunction, fertilization failure, and decrease in embryo competence (16, 39, 40). Since a large amount of ATP is consumed in the process of meiosis completion, fertilization, and embryonic development, it is possible that the decrease in ATP production due to deterioration of mitochondrial function results in a decrease in oocyte quality (41, 42). A study has shown that ATP contents ≧ 2 pmol/oocyte are necessary for oocytes to support normal embryo development (43). The reduced ATP production in oocyte may lead to a dysfunction of the spindle assembly checkpoint (SAC). A predominant mechanism of SAC silencing is dynein-mediated transport of certain kinetochore proteins along microtubules. ATP reduction prevents the release of dynein and its cargoes from the spindle poles and the redistribution of the core SAC proteins from attached kinetochores to spindle poles in metaphase-arrested cells, at a time when the SAC should be satisfied and silenced (44). The fidelity of SAC is compromised in aged oocytes, suggesting that SAC failure is a likely contributor to the increased incidence of chromosome abnormalities documented in oocytes and embryos of older women (45).

### Increased Vulnerability of Oocytes to Oxidative Stress

The efficacy of DNA double-strand break (DSB) repair mechanisms is attenuated in aged oocytes. Moreover, oocytes are acutely susceptible to accumulated DNA damage by reason of their extended prophase arrest. Increased oxidative damage brought about by mutations in mtDNA and the oxidative DNA repair enzyme OGG1 leads to accelerated aging phenotypes including spindle and chromosomal abnormalities in senescence-accelerated mice (46). An increase in the expression of the DNA DSB damage marker γH2AX in primordial follicles and germinal vesicle oocytes from aged mice and humans correlates with a decline in the expression of several DNA DSB repair genes including *Brca1, Mre11, Atm*, and *Rad51* (47). RNAi-mediated reduction of *Brca1* in oocytes results in abnormal spindle formation, chromosome misalignment, and a significant increase in hyperploid oocytes (48).

A global gene expression analysis of aged oocytes in mice revealed the decrease in mRNA expression of mitochondrial antioxidant genes, peroxiredoxin 3 (*Prdx3*) and thioredoxin 2 (*Txn2*), as well as cytosolic antioxidant genes, glutaredoxin 1 (*Glrx1*), glutathione S-transferase mu 2 (*Gstm2*), and superoxide dismutase 1 (*Sod1*) (49, 50). *Prdx3*, abundantly distributed in mitochondria, plays a key role as a regulator of mitochondrial H_2_O_2_ concentration and apoptosis (51). Another analysis has also reported reduced expression of Sod1 and Txn family members in MII oocytes from aged mice (48). SOD1 is highly expressed in human oocytes (52) and the addition of *Sod1* protein improves preimplantation development in mice (53). Embryos from SOD1-deficient mouse oocytes have significantly higher levels of superoxide than wild-type embryos and their preimplantation development is halted at the 2-cell stage under atmospheric oxygen. Instead of any treatments with antioxidants, only hypoxic culture with 1% O_2_ negated the 2-cell arrest (54). Interestingly, knockdown of either the cytoplasmic or mitochondrial SOD in Drosophila significantly increases the percentage of oocytes showing arm cohesion defects and provokes segregation errors (55). Chemical inhibition of SOD activity in porcine oocytes elicits a reduction in meiotic progression, decreased GSH levels, and diminished rates of cleavage and blastocyst formation (56). The depletion of GSH is also associated with altered spindle morphology, disturbed microtubule function, and chromosome clumping in hamster and bovine MII oocytes (57, 58). Furthermore, thioredoxins are also involved in the reduction and protection against oxidative stress-induced apoptosis, and the targeted mutation of *Txn1* causes embryonic lethality shortly after implantation (59). The decline in the fidelity of these protective mechanisms against OS collectively renders aged oocytes vulnerable to OS.

### OS Induces ER Stress and Dysfunction of Proteasome and Autophagy in Oocytes

OS is involved in the effect of aging on reproductive function by causing ER stress (60, 61). Acting as a major site for the biosynthesis of proteins, lipids and secretory proteins, the ER plays a key role in meeting the oocyte's increased demand for new proteins during oocyte maturation and embryo development. Therefore, ER stress and homeostasis play an important role in determining oocyte quality. OS can induce ER stress and an adaptive signaling cascade known as the unfolded protein response (UPR) by impeding correct protein folding and calcium homeostasis (62, 63). If the UPR-mediated response fails in correcting the protein-folding defect, apoptosis is activated. Interestingly, lycium barbarum polysaccharide (LBP), extracted from the traditional Chinese herbal medicine goji berry, has antioxidant and cryoprotective properties, and improves the developmental competence of mouse oocytes that were vitrified/warned at the germinal vesicle stage with cumulus cells (64). LBP may reduce ER stress, activate both PI3K/AKT and MAPK3/1, and prevent cell death (64).

There is also a theory that proteasome dysfunction due to accumulation of oxidatively induced damage of functional proteins is also involved in the deterioration of oocyte quality with age. An age-related decline in proteasome activity has been demonstrated in a multitude of mammalian tissues and cells (60–68). In fact, a comprehensive analysis revealed that in oocytes, many proteasome-related genes are expressed less with increasing age (50). Decreased proteasome activity in naturally aged mouse oocytes are positively correlated with increased protein modification by 4-hydroxynonenal (4-HNE), which is elevated by the lipid peroxidation chain reaction in conditions involving oxidative stress (65). An exposure of germinal vesicle oocytes to either H_2_O_2_ or 4-HNE contributes to decreased meiotic completion, increased spindle abnormalities, chromosome misalignments and aneuploidy (66).

Autophagy is an evolutionarily conserved phenomenon by which unwanted intracellular proteins and organelles are sequestered within autophagosomes and delivered to lysosomes for degradation. Autophagy was found as a cell survival mechanism in starving cells, and also has a role in cell death. It is generally accepted that autophagy induces ROS, but also reduces oxidative damage (69). Autophagy has an important role in removing damaged mitochondria by mitophagy and in reducing ER stress. Autophagy has been observed in mouse, rat, and porcine oocytes. In rat ovaries, all phases of the estrous cycle contain oocytes that simultaneously express proteins involved both in apoptosis and autophagy (67). Autophagy-defective oocytes, obtained from oocyte-specific *Atg5* knockout mice, could not develop beyond the four- and eight-cell stages after fertilization with *Atg5*-null sperm, suggesting a critical role of autophagy in pre-implantation mammalian development (68). In bovine embryos, a transient increase in autophagy also leads to decreased ER stress, which has a positive influence on *in vitro* preimplantation development (70). The consequences of autophagy modulation, including those that are mediated by OS during aging, may either promote cell survival or be associated with cell death (71). Decreased autophagy may provide a cellular environment allowing for the accumulation of dysfunctional mitochondria (72).

### Possible Role of the Sirtuin Family Against OS in Oocytes

The sirtuin family is involved in regulating the energy metabolism and stress resistance of mammalian cells (73). Recently, SIRT1 and SIRT3 have been revealed to have an important role as sensors and protectors of the redox balance in oocytes, granulosa cells, and early embryos (74). SIRT1 and SIRT2 have been found in both the nucleus and cytosol, while SIRT3, SIRT4, and SIRT5 have been detected exclusively in mitochondria; SIRT6 and SIRT7 have been localized only in the nuclear compartment (75–77). The antioxidant response, the “FoxO3a-MnSod axis” orchestrated by SIRT1, is attenuated with age in oocytes (78). Several studies have investigated anti-aging treatment with resveratrol or calorie restriction focusing on SIRT1 (69, 79). SIRT3 also acts in a protective role against stress conditions in preimplantation embryos (80). Decreased expression of SIRT3 correlates with lower embryonic developmental competence (81). Melatonin, which enhances SIRT1 and SIRT3 activity, is considered effective as a treatment for aging oocytes (81, 82).

### Adverse Effects of OS on Telomeres in Oocytes

OS and telomere shortening are correlated exponentially with aging of somatic cells (83). The ROS generated by compromised mitochondria could potentially oxidize proteins necessary for telomere maintenance (84). Telomeres lack protective proteins and sit in the nuclear membrane, where they are susceptible to lipid peroxidation (85). Intrinsically, their sequences are rich in guanine, which is quite susceptible to oxidation. Telomere shortening is also involved in failure of spindle formation, arrest of embryonic development, and fragmentation in oocytes (86). In an experiment administering a cigarette smoke condensate (CSC) to mouse 1-cell zygotes, OS induced chromosomal aberrations in mouse embryos via telomere shortening and loss, but the antioxidant N-acetyl-L-cysteine (NAC) prevented the defects induced by CSC (8). Fertilized mouse eggs treated with FCCP, which uncouples the mitochondrial electron transport pathway, also show significantly increased ROS and decreased developmental competence with telomere shortening and chromosomal fusion compared to control embryos (84). In reproductively aged mouse oocytes, Q-PCR and quantitative fluorescence *in situ* hybridization analyses show significant increase of OS and shortening of telomere lengths (6, 87). Human oocytes with shorter telomeres develop into more fragmented and more aneuploid preimplantation embryos with lower implantation rates (88, 89), whereas relative telomere length was comparable in aneuploid and euploid first polar bodies and blastomeres (90). Further, SIRT6, associated with oxidative homeostasis, has been identified as an important modulator of telomeres in age-related deterioration of mouse oocytes (91, 92). Overexpression of SIRT6 in oocytes from aged mice promotes telomere elongation in 2-cell embryos and lowers the incidence of apoptotic blastomeres (91). Further studies on the age-related alteration of telomere length in mammalian oocytes seem necessary.

## Conclusion

**Figure 1:**
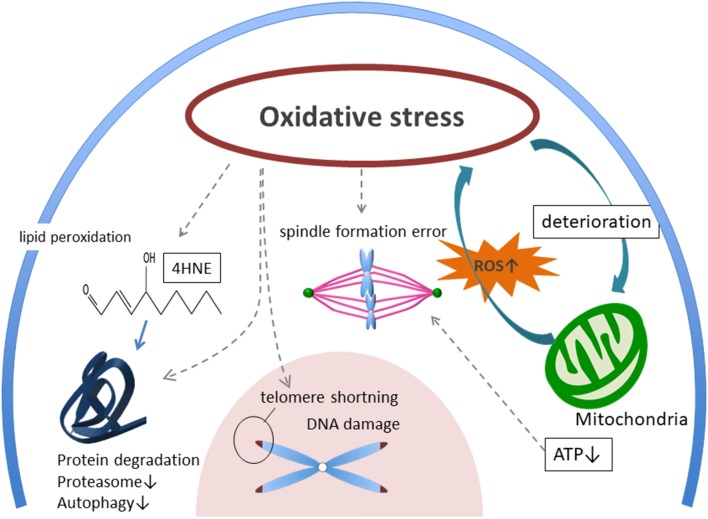
Possible mechanism of aged oocyte deterioration with accumulating oxidative stress. External ROS, AGEs, and accumulation of internal ROS from mitochondria are burdening oocytes as oxidative stress (OS). Then, OS induces deterioration of mitochondria, telomere shortening, spindle formation error, DNA damage, and protein degradation. ROS, reactive oxygen species. AGEs, glycation end-products. RAGE, receptor for advanced glycation end-products. 4-HNE, 4-hydroxynonenal.

In recent years, social progress of women has resulted in delayed reproduction. Reproductive medicine faces serious challenges because aging causes oocyte quality to deteriorate. This deterioration of oocyte quality is influenced by OS. OS damages many cellular components, including mitochondria, lipids, proteins, enzymes, and DNA, leading to ATP shortage, DNA break, chromosomal segregation error, dysregulation of autophagy and proteasome system (Figure 1). In particular, mitochondria are the most significant targets of OS as they are pivotal in controlling cell survival and death. Moreover, a theory showing DNA methylation and epigenetic errors as influencing OS on germ cells has also been proposed (50, 93, 94). However, direct evidence for a participation of OS in the aging process of human oocytes and mechanisms of protection against OS is still to be gathered.

## Author Contributions

HS and TH contributed to the drafting. All authors listed have made a substantial, direct and intellectual contribution to the work, and approved it for publication.

### Conflict of Interest

The authors declare that the research was conducted in the absence of any commercial or financial relationships that could be construed as a potential conflict of interest.
